# The value of optic nerve sheath measurement in patients presenting to the emergency department with hypertensive crisis

**DOI:** 10.1097/MD.0000000000042361

**Published:** 2025-05-02

**Authors:** Faruk Danış, Emre Kudu, Elif Öztürk İnce, Mehmet Ali Karaca, Bülent Erbil

**Affiliations:** a Department of Emergency Medicine, Hacettepe University Faculty of Medicine, Ankara, Türkiye; b Department of Emergency Medicine, Bolu Abant İzzet Baysal University Medical School, Bolu, Türkiye; c Department of Emergency Medicine, Marmara University Pendik Training and Research Hospital, İstanbul, Türkiye.

**Keywords:** emergency department, hypertensive crisis, optic nerve sheath diameter, ultrasonography

## Abstract

To evaluate whether bedside ultrasonographic measurement of optic nerve sheath diameter (ONSD) can assist in the diagnosis of hypertensive emergency and predict hospitalization in patients presenting to the emergency department (ED) with hypertensive crisis. This prospective observational study enrolled adult patients presenting with systolic BP > 180 mm Hg and/or diastolic BP > 120 mm Hg. ONSD was measured on admission and repeated after blood pressure was reduced to target levels. Diagnostic performance was assessed using ROC analysis. A total of 112 patients with hypertensive crisis were included, and 22.3% (n = 25) were diagnosed with hypertensive emergency. The mean ONSD in these patients (5.99 ± 0.65 mm) was significantly higher than in those with hypertensive urgency (5.11 ± 0.57 mm) (*P* < .001). Among 31 hospitalized patients, the mean ONSD (5.83 ± 0.71 mm) was also significantly higher than in those not hospitalized (5.11 ± 0.57 mm) (*P* < .001). ROC analysis showed good diagnostic performance for predicting hypertensive emergency, with the optimal ONSD cutoff identified as 5.8 mm (sensitivity: 68%, specificity: 93.1%, +LR: 9.86, −LR: 0.34). ONSD measurement is a rapid, noninvasive tool that may aid in early identification of hypertensive emergency and in predicting hospital admission. Its routine use in the ED could facilitate timely intervention and decision-making.

## 1. Introduction

A hypertensive emergency is defined as a severe elevation in blood pressure above 180 mm Hg systolic and/or 120 mm Hg diastolic, associated with new or worsening organ damage.^[1]^ In the event of hypertensive emergencies (hypertensive encephalopathy, intracranial hemorrhage, unstable angina pectoris, acute myocardial infarction, acute left heart failure with pulmonary edema, dissecting aortic aneurysm, preeclampsia, and eclampsia), blood pressure must be lowered immediately. Hypertensive urgencies (blood pressure > 180/110 mm Hg; papilledema, severe perioperative hypertension, and progressive target organ complications) are defined as situations in which blood pressure should be reduced to normal limits within a few hours.^[2]^ Early diagnosis and treatment are crucial when these patients when they present to the emergency department (ED).

Normal intracranial pressure (ICP) in adults is approximately 15 mm Hg, and values above 20 mm Hg it is considered elevated.^[3]^ Clinical manifestations of ICP increase usually appear late. Elevated ICP can be detected in the early period by optic nerve sheath diameter (ONSD) measurement, before it is seen in the patient’s physical examination. This can eliminate the undesired delay in diagnosis and treatment. It has been reported that some patients who presented to the ED with hypertensive crisis had elevated ICP.^[4,5]^ In hypertensive emergency patients, increased ICP can be detected in the early period and possible mortality and morbidity can be prevented.

Methods for ICP measurement are divided into invasive and noninvasive. Interventional methods are considered the gold standard for ICP measurement, but they are invasive. The subarachnoid space and retrobulbar segment are affected by ICP. Therefore, an increase in ICP can be predicted indirectly by measuring ONSD.^[6]^ The cheapest and most accessible method for predicting ICP increase is evaluation of ONSD by bedside ultrasonography (USG). There are many studies comparing ICP measurement by interventional applications with ultrasonographic ONSD measurement. When the specificity, sensitivity, positive or negative likelihood ratio, and diagnostic odds ratio were examined, no significant difference was found in either group.^[7,8]^ According to a meta-analysis by Duborg et al, the ONSD value showing an increase in ICP varied between studies with different cutoff values between 4.8 and 5.86 mm.^[7]^

In the present study, our aim was to determine the cutoff value of ONSD with ocular USG, which is easily accessible, inexpensive, and associated with minimal complication risk, and to guide the diagnosis and treatment in hypertensive emergency patients.

## 2. Materials and methods

### 2.1. Study design and setting

The study had a single-center, prospective, observational design. Approval was granted by Hacettepe University Clinical Research Ethics Committee (Approval no: 2019/11-26) and informed consent was obtained from all patients before inclusion.

The study was conducted in a tertiary university ED with approximately 100,000 patient admissions per year. In order to standardize the ONSD evaluation of all patients, ONSD was measured by a single senior (postgraduate year-four) emergency medicine resident who had an ultrasonography course certificate and sufficient experience in ONSD measurements.

### 2.2. Study population

Patients aged 18 years or older who presented to the ED between June 1, 2019, and June 1, 2020, with a SBP above 180 mm Hg and/or a diastolic blood pressure (DBP) above 120 mm Hg, who were diagnosed with hypertensive crisis (either hypertensive emergency or urgency), who were able to undergo bedside ocular ultrasonography, and who provided informed consent were included in the study. Patients with any known ocular or intracranial pathology that could interfere with the accuracy of ONSD measurement – such as hydrocephalus, ventriculoperitoneal shunt, intracranial mass, pseudotumor cerebri syndrome, Graves’ disease, or sarcoidosis – were not included. Similarly, those who could not undergo ultrasonography due to facial trauma, lack of cooperation, or technical limitations, as well as individuals who did not complete the required study procedures, were also not included. In addition, some patients who were initially enrolled in the study were later excluded from the final analysis due to reasons such as withdrawal of consent, inability to complete ultrasonographic measurements during treatment, early departure from the ED before completion of clinical management, in-hospital transfer, or death.

### 2.3. Outcome measures

The primary outcome of the study was to determine the diagnostic accuracy of ONSD measurements for identifying hypertensive emergency in patients presenting with hypertensive crisis, and to establish an optimal ONSD cutoff value using receiver operating characteristic (ROC) analysis. Secondary outcomes included evaluating the relationship between ONSD and SBP at admission, as well as identifying an ONSD threshold value associated with the need for hospitalization.

### 2.4. Data collection methods

The demographic data of the participants and symptoms that may be associated with hypertension (headache, epistaxis, blurred vision, double vision, chest pain, shortness of breath, dizziness, nausea, abdominal pain, dizziness, pain or weakness in the extremities, and confusion) were recorded in case report forms.

**Figure 1.**
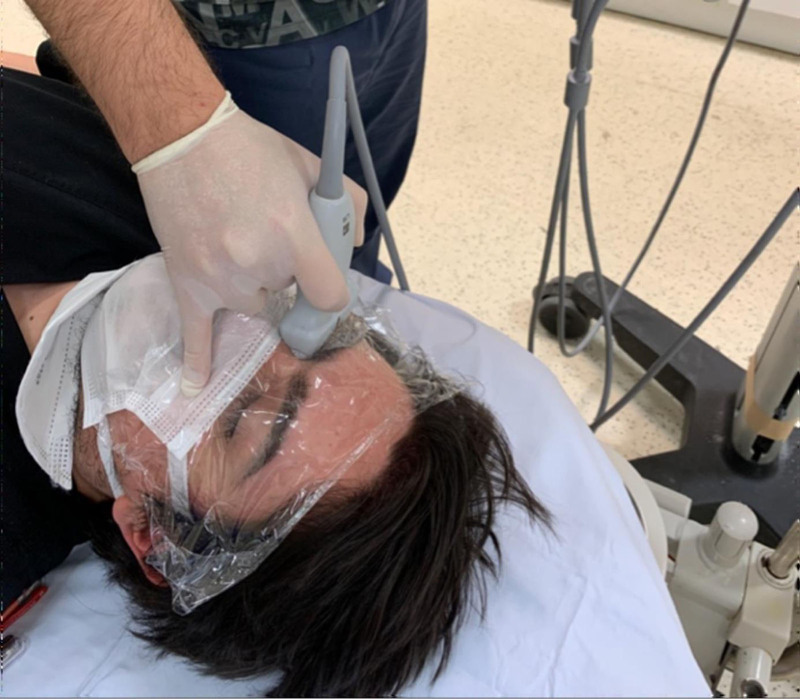
Bedside ultrasonographic measurement of the ONSD in a supine patient. A linear transducer is gently placed over the closed eyelid, with a stretch film barrier and ultrasound gel applied to avoid direct contact. ONSD = optic nerve sheath diameter.

Bedside ocular ultrasonographic measurement of the ONSD was performed without interfering with the patient’s clinical examination or treatment. All procedures were carried out using a 6 to 10 MHz linear probe (SonoSite Edge, SonoSite Inc., Bothell) in B-mode. Patients were positioned in the supine position on the examination stretcher. To avoid direct gel contact with the eyes, both eyelids were first covered with stretch film. Ultrasound gel was then applied on top of the film, and the linear probe was gently placed on the upper eyelid in a transverse orientation. Care was taken to avoid exerting any pressure on the globe during scanning (Fig. 1). The cursor was placed on the outer contours of the optic nerve 3 mm posterior to the papilla and the measurement was recorded. With this method, ONSD was measured twice in both eyes and the mean of all measurements was calculated and recorded in the case report form. In patients whose examination and treatment were ongoing, blood pressure and ONSD measurements were repeated 1 hour after systolic blood pressure (SBP) < 130 mm Hg and/or DBP < 80 mm Hg was reached and they were recorded in the case report form.

### 2.5. Statistical analysis

While calculating the sample size of our study, the table in the article entitled “Sample size estimation for correlations with pre-specified confidence interval” prepared by Murray Moinester and Ruth Gottfried was used.^[9]^ In the hypothesis of our study, the correlation coefficient was targeted as 0.70 (95% CI: 0.6–0.8) and the sample size in the table was 105. Based on this evaluation, the desired sample size for the study was approximately 115 after 10% was added (predicting possible data losses).

The statistical analyses were conducted using IBM SPSS for Mac Version 23.0 (Chicago). The Kolmogorov–Smirnov test and the Shapiro–Wilk *W* test were used to determine whether the numerical variables were normally distributed. These tests were applied to continuous variables such as ONSD values, systolic and DBP, and age. Numerical data normally distributed were shown as mean ± standard deviation, while numerical data not normally distributed were shown as median and interquartile range (IQR). Categorical variables were summarized as counts and percentages. The similarity of group variances was investigated with the Levene test. In the comparisons of 2 independent groups with continuous variable data, Student *t*-test was used for normally distributed data, while the Mann–Whitney *U* test was used for data not normally distributed. The paired *t*-test was used for normally distributed data and Wilcoxon test was used for non-normally distributed data in comparisons of 2 dependent groups with continuous variable data. The relationship between categorical variables was determined with Pearson chi-squared test. The cutoff point for ONSD values in demonstrating hospitalization was determined by ROC curve analysis. Sensitivity, specificity, +LR, and −LR for the best cutoff point were given. Youden Index was applied to identify the optimal cutoff point on the ROC curve by maximizing the sum of sensitivity and specificity. The area under the ROC curve was calculated. Spearman correlation analysis was performed on data that were not normally distributed in order to determine the correlation between 2 numerical variables. Point-biserial correlation analysis was performed to determine the correlation between a numerical variable and a true dichotomous variable. All statistical calculations were evaluated at the 95% confidence interval and *P* < .05 significance level.

## 3. Results

**Figure 2.**
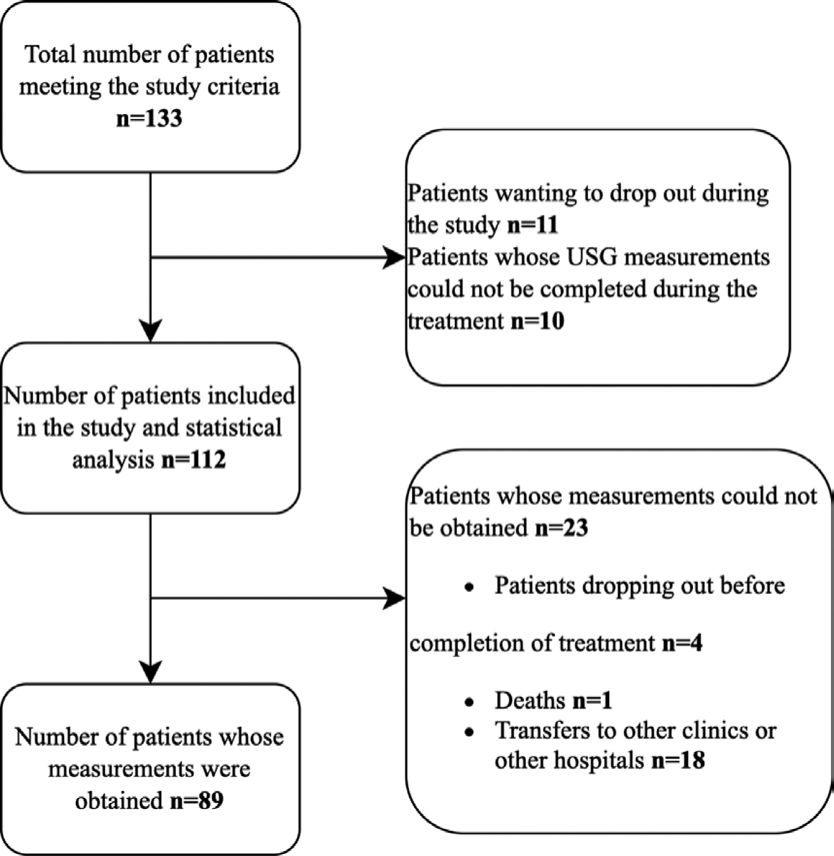
Flowchart showing patient inclusion, exclusion, and follow-up. Of 133 patients initially assessed, 112 were included in the analysis, and follow-up measurements were obtained from 89 patients. Reasons for exclusion and follow-up loss are detailed.

A total of 133 patients were assessed for eligibility during the study period. Of these, 21 patients were not included in the study due to not meeting the inclusion criteria or being unable to complete initial ultrasonographic evaluation. Consequently, 112 patients were included in the main statistical analysis. However, 23 of these patients left the ED before their blood pressure could be reduced to target levels, or were transferred to another facility, or died during early management. Therefore, follow-up ONSD measurements could not be obtained for these individuals, and they were not included in the follow-up analyses. As a result, follow-up evaluations were completed for 89 patients (Fig. 2).

Of the 112 patients included, 68 (60.7%) were female. Twenty-five (22.3%) patients were diagnosed with hypertensive emergency and 87 (77.7%) with hypertensive urgency. Among the 25 patients diagnosed with hypertensive emergency, the final diagnoses were as follows: acute ischemic stroke (n = 11), acute myocardial infarction (n = 7), aortic dissection (n = 4), subdural hematoma (n = 2), and acute renal failure (n = 1). Nine (20.5%) of the men and 16 (23.5%) of the women were diagnosed with hypertensive emergency.

The mean age of the patients diagnosed with hypertensive emergency (71 years, SD: 10.86; 95% CI: 66.48–75.44) was significantly higher than that of the patients diagnosed with hypertensive urgency (62 years, SD: 13.34; 95% CI: 59.19–64.88) (*P* = .003, t-test).

The mean age, sex distribution, comorbidities, and mean ONSD values of the patients are presented in Table 1.

**Table 1:** The patients’ demographic characteristics and mean ONSD.

Age (yr)	64.03 (SD: 13.31; 95% CI: 61.53–66.52)
Female	68 (61%)
Comorbidities	n (%)
Hypertension	68 (61%)
Diabetes	32 (29%)
Coronary artery disease	32 (29%)
Chronic obstructive pulmonary disease	4 (4%)
Asthma	4 (4%)
Congestive heart failure	6 (5%)
Mean ONSD (mm)	5.3 (SD: 0.69; 95% CI: 5.17–5.43)

CI = confidence interval, ONSD = optic nerve sheath diameter, SD = standard deviation.

The mean ONSD in the 88 patients who presented to the ED with at least 1 symptom that may be associated with hypertension at the time of admission was 5.36 mm (SD: 0.74; 95% CI: 5.21–5.52) and in the 24 who presented without any of these symptoms at the time of admission was 5.11 mm (SD: 0.41, 95% CI: 4.93–5.23). The mean difference between the 2 groups was 0.25 mm, which is significant (*P* = .031; 95% CI: 0.02–0.5). The mean ONSD values according to the complaint at presentation are given below (Table 2).

**Table 2:** Comparison of the mean values of ONSD according to the complaints of the patients.

		Number of patients	ONSD at presentation (mean ± SD, mm)	*P*-value*
High blood pressure†	Yes	29	5.17 ± 0.46	.114
	No	83	5.35 ± 0.76	
Headache	Yes	27	5.28 ± 0.13	.78
	No	85	5.32 ± 0.76	
Dizziness	Yes	7	5.79 ± 0.62	.059
	No	105	5.28 ± 0.69	
Chest pain	Yes	16	5.31 ± 0.61	.965
	No	96	5.31 ± 0.70	
Shortness of breath	Yes	11	5.59 ± 0.29	**.01**
	No	101	5.27 ± 0.71	
Nosebleed (median [IQR])	Yes	5	4.8 (0.3)	.068**
	No	107	5.35 (0.85)	
Pain or weakness in the extremities	Yes	22	5.03 ± 0.8	**.032**
	No	90	5.38 ± 0.65	
Nausea	Yes	6	5.01 ± 0.6	.276
	No	106	5.32 ± 0.69	
Stomach ache	Yes	3	4.16 ± 0.73	**.003**
	No	109	5.33 ± 0.67	
Feeling dazed	Yes	37	5.21 ± 0.44	.216
	No	75	5.35 ± 0.78	
Confusion	Yes	18	5.86 ± 0.74	**<.001**
	No	94	5.20 ± 0.63	
At least 1 HT-associated symptom	Yes	88	5.36 ± 0.74	**.031**
	No	24	5.11 ± 0.41	

Statistically significant *P*-values are written in bold.

HT = hypertension, IQR = interquartile range, ONSD = optic nerve sheath diameter, SD = standard deviation.

† Patients who presented to the emergency department because of high blood pressure measurement.

* *t*-test.

** Mann–Whitney *U* test.

The mean ONSD of all patients on presentation to the ED was 5.30 mm (SD: 0.69; 95% CI: 5.17–5.43). The mean ONSD of the patients diagnosed with hypertensive emergency at admission (5.99 ± 0.65 mm) was significantly higher than that of the patients diagnosed with hypertensive urgency (5.11 ± 0.57 mm) (*P* < .001). Moreover, the mean ONSD of the 31 patients who needed hospitalization at admission (5.83 ± 0.71 mm) was significantly higher than that of the 81 patients who did not (5.11 ± 0.57 mm) (*P* < .001) (Table 3).

**Table 3:** ONSD values according to the patients’ diagnoses and hospitalization needs.

	ONSD at presentation (mean ± SD, mm)	*P*-value*	95% Confidence interval of the difference
Hypertensive emergency (n = 25)	5.99 ± 0.65	<.001	0.62 to 1.15
Hypertensive urgency (n = 87)	5.11 ± 0.57		
Required hospitalization (n = 31)	5.83 ± 0.71	<.001	0.47 to 0.98
Did not require hospitalization (n = 81)	5.11 ± 0.57		

CI = confidence interval, ONSD = optic nerve sheath diameter, SD = standard deviation.

* *t*-test.

**Figure 3.**
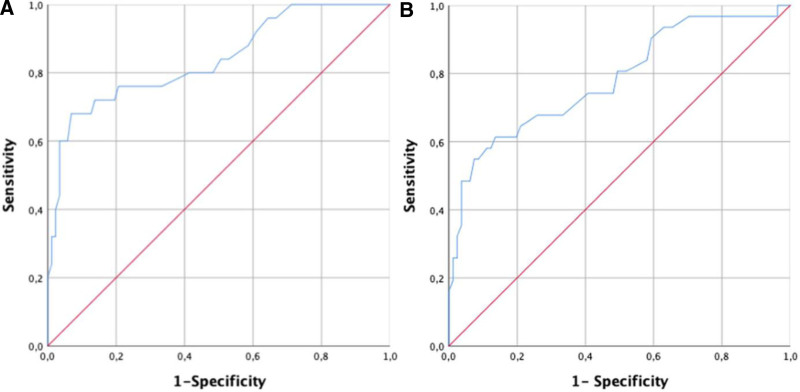
Receiver operating characteristic (ROC) curves for the diagnostic performance of optic nerve sheath diameter (ONSD): (A) prediction of hypertensive emergency. (B) Prediction of hospitalization.

When the ONSDs of the patients at the time of admission were evaluated with the ROC curve in terms of predicting hypertensive emergency, the area under the curve was 0.839 and so the success was good (*P* < .001; 95% CI: 0.74–0.94) (Fig. 3A). According to the analysis, the highest sensitivity and specificity values (Youden index) in predicting the hypertensive emergency were achieved when the ONSD threshold value was 5.8 mm [sensitivity: 68% (95% CI: 46.5–85.1); specificity: 93.1% (95% CI: 85.6–97.4); +LR: 9.86 (95% CI: 4.4–22.3), −LR: 0.34 (95% CI: 0.2–0.6)].

Evaluation of the hospitalization requirement of the patients revealed that 31 required hospitalization, while 81 were discharged from the ED. When the ONSDs of the patients at the time of admission were evaluated with the ROC curve in terms of predicting the need for hospitalization, the area under the curve was 0.783 (*P* < .001; 95% CI: 0.68–0.88) (Fig. 3B). This shows that the success of the test is moderate. According to the analysis, the highest sensitivity and specificity values (Youden index) in showing hospitalization were achieved when the ONSD threshold value was 5.6 mm [sensitivity: 61.29% (95% CI: 42.2–78.2); specificity: 86.42% (95% CI: 77–93); +LR: 4.51 (95% CI: 2.4–8.4), −LR: 0.45 (95% CI: 0.3–0.7)].

Evaluation of the 89 patients included in the study whose blood pressure was measured 1 hour after the target blood pressure was reached showed a significant difference between the ONSD measured at presentation and 1 hour after the target blood pressure was reached in those diagnosed with hypertensive urgency. Among these patients, there was also a significant difference between the ONSD measurements performed at presentation and 1 hour after reaching the target blood pressure in those diagnosed with hypertensive emergency (Table 4).

**Table 4:** Comparison of admission to the emergency department and follow-up ONSD measurements between the hypertensive urgency and emergency diagnosis subgroups.

Diagnosis	ONSD (mean ± SD, mm)		*P*-value*
	Presentation (0 hours)	Follow-up (1 hour)	
Hypertensive emergency (n = 8)	5.7 ± 0.68	5.26 ± 0.47	**.009 (95% CI: 0.15–0.72**)
Hypertensive urgency (mm) (n = 81)	5.09 ± 0.58	4.85 ± 0.46	**<.001 (95% CI: 0.18–0.3**)

Statistically significant *P*-values are written in bold.

CI = confidence interval, ONSD = optic nerve sheath diameter, SD = standard deviation.

* Paired *t*-test.

A weak positive correlation was found between presentation ONSD values and SBP in all patients (*R* = 0.241, *P* = .01, Spearman correlation analysis). Accordingly, 6% of the change in ONSD was associated with SBP. When this correlation was examined in the hypertensive emergency and urgency subgroups, no correlation was found between the presentation ONSD values of the patients diagnosed with hypertensive emergency and SBP (*P* = .144, Spearman correlation analysis). However, a moderate positive correlation was found between the presentation ONSD values of patients diagnosed with hypertensive urgency and SBP (*R* = 0.46, *P* < .001, Spearman correlation analysis). Accordingly, 21% of the change in the ONSD of patients diagnosed with hypertensive urgency was associated with SBP. There was no correlation between the presentation ONSD values of all patients and DBP (*P* = .05, Spearman correlation analysis).

Our study showed a moderate positive correlation between the presentation ONSD values of all patients and a diagnosis of hypertensive emergency or urgency (*R* = 0.489, *P* < .001, Spearman correlation analysis).

## 4. Discussion

Interventional methods are the gold standard for ICP measurement. However, since they involve measurement with intracranial catheters, complications such as hemorrhage (1.1%–5.8%), infection (15%), and loss of functionality of the catheter (6.3%–40%) may occur. In addition, this procedure cannot be performed if coagulopathy or thrombocytopenia is present.^[10]^ Computed tomography (CT), magnetic resonance imaging (MRI), and electroencephalogram (EEG) are the leading noninvasive methods. However, as these methods are expensive, difficult to access, and time-consuming, their usability is limited.^[11,12]^ Another noninvasive method is USG. With bedside ocular USG evaluation, ONSD can be measured quickly and non-invasively without exposing the patient to radiation or the risks involved in being transported from the emergency room. In the ED ultrasonography guide published by the American College of Emergency Medicine, ocular ultrasonography used in ONSD measurement is among the core applications.^[13]^ It has been reported that ONSD measurements performed by emergency physicians after 5 minutes of ocular ultrasonography training are successful when compared to those obtained by cranial MRI imaging.^[14]^ Studies examining the relationship between ONSD and ICP have shown a correlation between them and that ONSD increases with an increase in ICP.^[4,5,15,16]^ In the studies concerning hypertension and ONSD in the ED, even though there was no evaluation of the relationship between bedside USG measurements and the prediction of hypertensive emergency or between ONSD measurements and the prediction of need for hospitalization, we evaluated these relationships in our study.

Roque et al and Dikmetaş et al found a weak positive correlation between both SBP and DBP values and ONSD in hypertensive patients.^[4,5]^ In our study, we found a moderate positive correlation between SBP and ONSD in patients diagnosed with hypertensive urgency. According to the findings of our study, 21% of the changes in the ONSD of patients diagnosed with hypertensive urgency were associated with SBP (*R* = 0.46, *P* < .001, Spearman correlation analysis).

Early warning systems in patients presenting with hypertensive crisis may help the clinician during their examination and treatment. The results of our study showed that the detection of an ONSD of 5.8 mm and higher was successful in predicting hypertensive emergencies. For most hypertensive emergencies, mean arterial pressure should be reduced by about 10% to 20% in the first hour. This reveals the importance of early diagnosis. An ONSD of 5.8 mm or higher at presentation may serve as an early warning for the clinician during the examination and treatment and show that the treatment should be more aggressive.

Our study showed that mean ONSD at presentation to the ED in the patients diagnosed with hypertensive emergency was significantly higher than that of the patients diagnosed with hypertensive urgency (5.99 mm and 5.11 mm, respectively, *P* < .001). Hypertensive emergency is a rare and challenging condition to diagnose. Elevated ONSD values may support early identification of such cases in patients presenting with hypertensive crisis.

Dikmetaş et al found no significant difference in ONSD between symptomatic and asymptomatic hypertensive patients (0.527 mm and 0.523 mm, respectively, *P* = .254).^[5]^ Contrary to their study, we found that the mean ONSD in the patients who presented to the ED with at least 1 symptom that may be associated with hypertension was significantly higher (5.36 mm and 5.11 mm, respectively) (*P* = .031; 95% CI: 0.02–0.5). This difference may have been due to the different blood pressure threshold values of the patient groups included. While the threshold blood pressure value was 180/120 mm Hg for the patient groups included in our study, in Dikmetaş et al study the threshold value was 140/90 mm Hg.

Setting criteria for hospitalization and discharge in the ED ensures safe management of patients. In our study, the mean ONSD of the patients who required hospitalization was significantly higher than the mean of those who did not (5.83 mm and 5.11 mm, respectively, *P* < .001). Another important finding of our study was that 5.6 mm and higher ONSD measurements are valuable in assessing the need for hospitalization. An ONSD of 5.6 mm or higher at presentation to the ED can be a guide for clinicians in terms of predicting the need for hospitalization.

In hypertensive emergency, target organ damage requires intravenous therapy and hospitalization. To establish the presence of target organ damage, history and physical examination, as well as imaging methods such as electrocardiogram, chest X-ray, serum creatinine, urinalysis, cardiac biomarkers, CT, and MRI are often needed. However, these methods may take time and delay decision-making in the ED. Using the patients’ ONSD values for this purpose may help shorten that time and guide early intervention.

Our results suggest that high ONSD values can assist clinicians in determining whether the patient is symptomatic, in diagnosing hypertensive emergency, and in predicting the need for hospitalization. In particular, an ONSD value ≥ 5.8 mm at ED presentation may serve as a practical early warning indicator. As a rapid, bedside, and noninvasive method, ONSD measurement can support emergency physicians in identifying high-risk patients and in making timely decisions regarding imaging, monitoring, or hospitalization.

This study has several limitations. First, follow-up ONSD measurements could not be performed in 23 patients who left the ED before achieving the target blood pressure, which prevented longitudinal evaluation in those cases. Second, as a single-center study, the generalizability of the findings to other emergency care settings may be limited. Third, all ONSD measurements were performed by a single trained operator, which precluded interobserver variability analysis. Fourth, the sample size – particularly for patients diagnosed with hypertensive emergency – was relatively small, which may limit the statistical power of subgroup analyses.

## 5. Conclusion

Bedside ultrasonographic measurement of ONSD appears to be a practical, rapid, and noninvasive tool that may assist emergency physicians in the early identification of hypertensive emergency. Its use in the ED could support timely decision-making, including risk stratification and disposition planning, in patients presenting with hypertensive crisis. Incorporating ONSD measurement into the initial assessment may improve the efficiency of clinical evaluation and contribute to better patient outcomes.

## Author contributions

**Conceptualization:** Faruk Daniş, Bülent Erbil.

**Data curation:** Faruk Daniş.

**Formal analysis:** Faruk Daniş.

**Investigation:** Faruk Daniş, Emre Kudu.

**Methodology:** Faruk Daniş, Bülent Erbil.

**Project administration:** Faruk Daniş.

**Software:** Faruk Daniş, Emre Kudu.

**Supervision:** Bülent Erbil.

**Visualization:** Faruk Daniş, Emre Kudu.

**Writing – original draft:** Faruk Daniş, Emre Kudu, Elif Öztürk İnce, Mehmet Ali Karaca, Bülent Erbil.

**Writing – review & editing:** Faruk Daniş, Emre Kudu, Elif Öztürk İnce, Mehmet Ali Karaca, Bülent Erbil.
